# How Awareness Changes the Relative Weights of Evidence During Human Decision-Making

**DOI:** 10.1371/journal.pbio.1001203

**Published:** 2011-11-22

**Authors:** Floris P. de Lange, Simon van Gaal, Victor A. F. Lamme, Stanislas Dehaene

**Affiliations:** 1 Donders Institute for Brain, Cognition and Behavior, Radboud University Nijmegen, Nijmegen, the Netherlands; 2 Department of Psychology, University of Amsterdam, Amsterdam, the Netherlands; 3 Inserm, Cognitive Neuroimaging Unit, Gif-sur-Yvette, France; 4 Commissarìat à l'Energie Atomique, Neurospin Center, Gif-sur-Yvette, France; 5 Cognitive Science Center Amsterdam, the Netherlands; 6 Université Paris-Sud 11, Orsay, France; 7 Collège de France, Paris, France; University of Oregon, United States of America

## Abstract

Human decisions are based on accumulating evidence over time for different options. Here we ask a simple question: How is the accumulation of evidence affected by the level of awareness of the information? We examined the influence of awareness on decision-making using combined behavioral methods and magneto-encephalography (MEG). Participants were required to make decisions by accumulating evidence over a series of visually presented arrow stimuli whose visibility was modulated by masking. Behavioral results showed that participants could accumulate evidence under both high and low visibility. However, a top-down strategic modulation of the flow of incoming evidence was only present for stimuli with high visibility: once enough evidence had been accrued, participants strategically reduced the impact of new incoming stimuli. Also, decision-making speed and confidence were strongly modulated by the strength of the evidence for high-visible but not low-visible evidence, even though direct priming effects were identical for both types of stimuli. Neural recordings revealed that, while initial perceptual processing was independent of visibility, there was stronger top-down amplification for stimuli with high visibility than low visibility. Furthermore, neural markers of evidence accumulation over occipito-parietal cortex showed a strategic bias only for highly visible sensory information, speeding up processing and reducing neural computations related to the decision process. Our results indicate that the level of awareness of information changes decision-making: while accumulation of evidence already exists under low visibility conditions, high visibility allows evidence to be accumulated up to a higher level, leading to important strategical top-down changes in decision-making. Our results therefore suggest a potential role of awareness in deploying flexible strategies for biasing information acquisition in line with one's expectations and goals.

## Abstract 2

A combined behavioral and brain imaging study shows how sensory awareness and stimulus visibility can influence the dynamics of decision-making in humans.

## Author Summary

When making a decision, we gather evidence for the different options and ultimately choose on the basis of the accumulated evidence. A fundamental question is whether and how conscious awareness of the evidence changes this decision-making process. Here, we examined the influence of sensory awareness on decision-making using behavioral studies and magneto-encephalographic recordings in human participants. In our task, participants had to indicate the prevailing direction of five arrows presented on a screen that each pointed either left or right, and in different trials these arrows were either easy to see (high visibility) or difficult to see (low visibility). Behavioral and neural recordings show that evidence accumulation changed from a linear to a non-linear integration strategy with increasing stimulus visibility. In particular, the impact of later evidence was reduced when more evidence had been accrued, but only for highly visible information. By contrast, barely perceptible arrows contributed equally to a decision because participants needed to continue to accumulate evidence in order to make an accurate decision. These results suggest that consciousness may play a role in decision-making by biasing the accumulation of new evidence.

## Introduction

Many decisions can be formalized as a process of accumulation of evidence over time, ultimately favoring one alternative over another [1],[2]. Evidence accumulation models have successfully captured the neural dynamics of simple decisions in a visual motion categorization task [3] as well as more complex decisions in which discrete pieces of evidence need to be integrated [4]. Here we investigate whether accumulation of evidence is affected by the level of awareness of the information.

Visual subliminal priming studies have shown that perceptual [5]–[7], cognitive [8],[9], motor [10], and executive [11],[12] stages can all be influenced by subliminal information. Furthermore, the amount of priming and subliminal processing increases linearly with prime processing [13],[14], suggesting that some stages of evidence accumulation can proceed without awareness. Also, relatively long-term effects of subliminal priming have sometimes been observed [15]–[18], suggesting that accumulation of unconscious information is possible. However, it is an open question whether and how awareness modulates the way evidence is accumulated during decision-making.

Contemporary models of subliminal information processing posit that subliminal information is marked by a lack of “global ignition” [19], meaning that it cannot enter into a global workspace system that allows it to be held in working memory and broadcasted to a variety of higher level neural processors. This lack of ignition may preclude “access” to the information. Therefore, awareness may be a necessary condition for biasing and modifying the sensory evidence in line with one's expectations and goals during decision-making.

In this study, we directly test the potential role of awareness in human decision-making using a previously described task in which participants have to accumulate sequentially presented pieces of evidence across an extended period of time. We previously observed a dependency of evidence accumulation on the amount of prior accumulated evidence: when prior evidence was already strong, participants weighted the newly incoming information much less than when prior evidence was weak and indecisive [20]. Here we hypothesize that this top-down modulation may depend on awareness. While accumulation may be possible irrespective of the level of awareness [15]–[18], it may appear qualitatively different depending on awareness level. Specifically, if awareness is necessary for top-down biasing of information during decision-making, low-visible evidence may be accumulated in a linear fashion, i.e. adding and subtracting new information without regard to the history of prior accumulated evidence. Non-linearities in evidence accumulation (e.g., giving less weight to new information under conditions of high certainty [20],[21]), which are a more optimal decision strategy within a Bayesian decision-making framework [22], may be present only for high-visible evidence.

**Figure 1:**
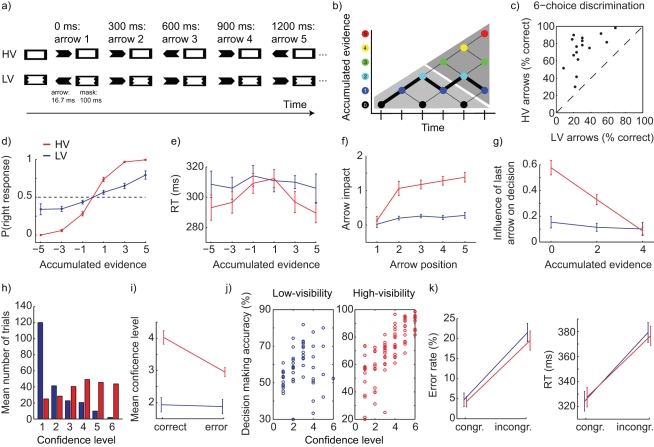
Task set-up and behavioral performance. (A) Task. Participants were shown a sequence of five arrows that were briefly shown and subsequently masked. Masks were constructed such that the arrows were either highly visible (HV) or near the threshold of awareness (low-visibility, LV). Participants had to decide whether the predominant direction of the arrow sequence was left or right. (B) Evidence accumulation diagram. Participants start with no evidence for either direction. Each incoming arrow moves the sum up or down in the diagram. Solid lines show the transitions for the HV example in panel A. States at which no decision can yet be made are highlighted in light-grey; states at which enough evidence is available for the decision are highlighted in dark-grey. (C) Individual performance during the six-choice discrimination task (Experiment 2) for HV and LV stimuli. (D) Decision-making performance as a function of accumulated evidence for HV and LV stimuli. Negative and positive numbers denote evidence for a left and right response, respectively (number of right-arrows minus number of left-arrows). (E) Reaction times as a function of accumulated evidence for HV and LV stimuli. (F) Impact of each arrow on the final decision (i.e., the extent to which the direction of the arrow determined the decision) over time for HV and LV trials. (G) Influence of the last arrow on the final decision as a function of the amount of previously accumulated evidence for HV and LV stimuli. (H) Confidence ratings of visibility for HV and LV arrows (Experiment 3). (I) Confidence ratings for correct and error trials for HV and LV arrows (Experiment 3). (J) Relationship between confidence level and performance for HV and LV stimuli (Experiment 3). (K) Priming strength of single HV or LV arrow as measured in a distinct masked priming task, in terms of error rate (left panel) and reaction times (right panel, Experiment 4).

We tested this hypothesis by using a decision-making task in which a sequence of five arrows was presented at either high or low visibility (HV versus LV), by means of masking (Figure 1A). In a series of behavioral experiments, we established whether and how evidence is accumulated over time, depending on the level of awareness. We also assessed the relationship between accumulated evidence and subjective decision confidence for stimuli at both awareness levels. Finally, we tracked accumulation-related neural activity over time in the human brain for both types of information, using magneto-encephalography (MEG).

## Results

### Behavioral Markers of Evidence Accumulation Over Time

On each trial, participants (*N* = 16) were presented with a stream of five arrows, each of which could point to the left or right with equal probability. Participants had to quickly decide on the overall direction of the arrows by pressing a button at the end of each stream with their left or right index finger, guessing if necessary (Figure 1A). Strength of the evidence could range from one (low evidence, e.g. two left and three right arrows) to five (high evidence, e.g. five right arrows, see evidence accumulation diagram in Figure 1B). Visibility of the arrows was manipulated by masking them with an effective “metacontrast” mask (leading to low visibility, LV) or with an equiluminant but less effective “pseudo” mask (leading to high visibility, HV; see Figure S1 for details) [14],[23]. On each trial, all arrows were either LV or HV. Stimulus and mask duration were identical for LV and HV conditions, allowing us to compare behavioral performance of evidence accumulation and the underlying neural responses without confounding stimulus visibility with basic task parameters [24]. A six-choice discrimination task performed after the main experiment confirmed that visibility of the arrows was much poorer on LV trials than on HV trials (31% correct versus 73% correct, Figure 1C; difference: *p*<0.001, see Materials and Methods for further details).

In the decision-making task, behavioral performance was less accurate for LV than for HV trials (60% versus 81% correct, *p*<0.001). Nevertheless, for both LV and HV trials, performance increased with increasing amount of evidence (both *p*<0.001), and thus evidence was accumulated for both trial types. For HV trials, performance approached a ceiling level of 100% correct for the highest evidence levels. For LV trials, the increase in performance was linear, peaking at 73% correct on trials with five identical arrows (Figure 1D). There was a trend of a right-side bias for LV trials when all arrows pointed in the same direction (67% versus 79%: T = 1.78, *p* = 0.096). Although we had no a priori hypothesis for such a bias, the finding is in line with earlier psychophysical studies showing that choices can be strongly biased by hand-preference especially under conditions of uncertainty (as in the case of the LV stimuli) [25]–[27].

While evidence accumulation was present for both LV and HV stimuli, there were striking behavioral differences in terms of *how* evidence was accumulated between conditions. First, the speed of decision-making was modulated by the amount of accumulated evidence for HV (*p*<0.001) but not for LV information (*p* = 0.31), leading to a significant interaction (*p* = 0.025, Figure 1E). Second, the “impact” of each successive arrow on the final decision varied as a function of time and accumulated evidence only for HV trials. We defined the impact of an arrow as the extent to which the arrow changed the response proportion in the direction of the arrow (see Materials and Methods for details on the exact quantification procedure). We observed a monotonically increasing impact of arrows on the decision as a function of time for HV stimuli (*p*<0.001), while this modulation of time was only marginal for LV stimuli (*p* = 0.07), leading to a significant interaction (*p*<0.001, Figure 1F). Moreover, for HV arrows, the influence of the last arrow, defined as the extent to which it changed the response probability in the direction of the arrow, decreased linearly with the amount of previously accumulated evidence: the larger the amount of accumulated evidence, the less influence the last arrow had on the decision (*p*<0.001), as expected from a rational strategy of progressively disregarding the arrows once sufficient evidence is obtained (Figure 1B). This modulation of accumulation by prior evidence was absent for LV stimuli (*p* = 0.44), leading to a significant interaction (*p*<0.001, Figure 1G).

Together, these results show that strategic effects on decision-making strongly depend on the awareness level of the stimuli. Interestingly, these results were not simply due to the possibility that, during HV trials, participants stopped performing the task after having observed a sufficient amount of arrows. A “logical counting” algorithm would not give any weight to the last arrow when two or four pieces of evidence had already been accumulated, since the last arrow cannot change the decision anymore. In our data, however, the last arrow did have a sizeable influence on the decision even when four pieces of evidence had already been accumulated (Figure 1G, red line, right data point).

We further explored the relationship between decision-making performance and subjective confidence in a new group of 16 participants; this time we additionally asked them to rate their confidence of having responded correctly after every trial on a 6-point scale (1 = pure guess, 6 = 100% sure). Overall decision-making performance was similar as in the MEG environment (in terms of overall performance and increase in performance with increasing evidence). As expected, overall confidence for LV arrows was much lower than for HV arrows (1.9 versus 3.8: *p*<0.001; Figure 1H). More interestingly, participants' confidence level for correct responses and incorrect responses was nearly equal for LV arrows (difference = 0.05, *p* = 0.093), but strongly dissociated for HV arrows (difference = 1.07, *p*<0.001; Figure 1I), resulting in a significant interaction (*p*<0.001). Thus participants had little insight in their accuracy level when arrows were strongly masked (LV), but they could very well distinguish correct from error trials when arrows were only weakly masked (HV). Since the inability to perform second-order confidence judgments has been proposed as a marker of lack of awareness [28], the results confirm that awareness was strongly reduced on LV compared to HV trials. When directly correlating decision-making performance and confidence for the correct trials, there was a strong correlation between these measures for HV trials (r = 0.77, *p*<0.001), while there was only a weak and marginally significant correlation for LV arrows (r = 0.23, *p* = 0.087, Figure 1J). However, this was likely due to the restricted range of confidence during LV since most participants reported low confidence levels for LV trials. When the range was restricted to the lowest three confidence levels, there was in fact a significant correlation between decision-making performance and confidence also for LV trials (r = 0.66, *p*<0.001). Overall, the results revealed that participants had markedly reduced confidence in their decision making for LV arrows, but could still use the information to achieve above-chance performance on the decision task.

Finally, we tested whether there was a difference in “stimulus strength” between the LV and HV arrows. In all experiments described so far, the arrow stimuli themselves were identical between conditions, and the only difference was the efficacy of the mask. Therefore, theoretically, the bottom-up stimulus strength, i.e. the ability of the stimulus to automatically climb up the sensorimotor pathways, may be equal for both conditions, even though visibility was strongly dissociated. To test this notion directly, we assessed and directly compared the priming strength of the LV and HV stimuli. Sixteen participants performed a simple masked priming experiment in which they responded as fast as possible to the direction of the mask (whose external outline was changed to a left- or right-pointing arrow; see Figure S1 and [14]). The mask was preceded by a LV or HV prime arrow pointing into the same or the opposite direction as the target. Congruence of the arrow prime resulted in significantly shorter reaction times (RT) and lower error rates (ER) to the mask for both LV and HV primes (all *p*<0.001). Crucially, this priming effect was not significantly different between LV and HV primes (RT priming effect: LV = 55 ms, HV = 50 ms, *p* = 0.13; ER priming effect: LV = 18%, HV = 17%, *p* = 0.39; Figure 1K), in line with earlier findings [29]. This shows that the bottom-up stimulus strength is equal for LV and HV arrows, and points to an interesting dissociation between the “direct” priming impact of a stimulus and its visibility and its long-term effect on decision-making.

### Neural Markers of Visibility

**Figure 2:**
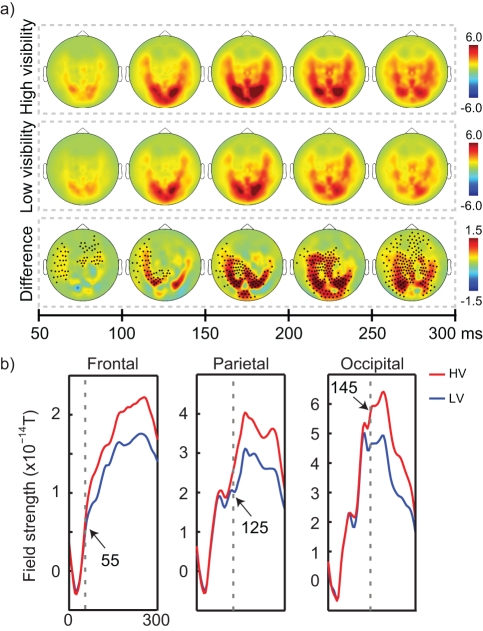
Neural markers of visibility. (A) Topographical representation of overall activity engendered by HV (top row) and LV (middle row) arrows, as well as differences in activity between them (bottom row). Activity (differences) is shown at five intervals after the onset of the arrow, from 50–300 ms. Dots indicate significant clusters of larger activity for HV than LV stimuli (*p*<0.05, corrected for multiple comparisons). (B) Time course of fronto-central (left panel), parietal (middle panel), and occipital (right panel) clusters for HV and LV arrows. Time course of frontal activity was plotted for all sensors with significant differences in the 50–100 ms interval, the parietal activity time course for the significant parietal sensors in the 100–150 ms interval, and occipital activity for the significant occipital sensors in the 150–200 ms interval. The dotted lines and numbers indicate the latency (in ms) at which differences between conditions first became significant.

Using MEG, we first investigated whether LV and HV arrows were processed differently in the human brain, irrespective of evidence or direction. We used a cluster-level statistic to establish the significance of differences between conditions. This method effectively controls the type I error rate in situations involving multiple comparisons (such as 275 sensors) by clustering neighboring sensor pairs that exhibit the same effect (see Materials and Methods for more details). A direct comparison of LV and HV arrows, collapsed across all five arrows, revealed that there was larger activity for HV than LV arrows over left frontal and fronto-central sensors (50–100 ms interval, *p*_cluster_ = 0.018 and *p*_cluster_ = 0.016, respectively). At a later interval, there was larger activity for HV than LV arrows over parietal (100–150 ms interval, *p*_cluster_ = 0.001) and occipital (150–300 ms interval, *p*_cluster_<0.001; Figure 2) sensors. A detailed time course analysis estimated the first point of significant difference at 55 ms for the frontal cluster, 125 ms for the parietal cluster, and 145 ms for the occipital cluster (Figure 2B). The early frontal difference between HV and LV arrows was present for all arrows except for the first arrow of the sequence (Figure S2A), while the occipital and parietal amplification for HV arrows was visible for each and every arrow (Figure S2B,C). Interestingly, there was a behavioral counterpart of the frontal asymmetry between the first and subsequent arrows: whereas the first arrow had equal effects on the decision for LV and HV arrows, there were large differences in the weight of the subsequent arrows on the decision (Figure 1F).

### Neural Markers of Evidence Accumulation Over Time

Previously we identified an inverse relationship between parietal and central neural activity and the amount of accumulated evidence: when more evidence was accumulated, neural activity evoked by new incoming stimuli was attenuated [20] (see [30],[31] for comparable results). This pattern is consistent with the strategy to reduce the weight of new evidence once substantial evidence has already been accumulated. Behavioral results indeed showed that the impact of the last arrow decreased with the total amount of previously accumulated evidence, but for HV arrows only (Figure 1G).

For the analysis of evidence accumulation in the MEG environment, we compared activity for LV and HV arrows that had “low prior accumulated evidence” and “high prior accumulated evidence,” averaged across the third to fifth arrow presentation (the first two arrow presentations are not taken into account since there is no differential amount of prior accumulated evidence until after the first two arrows have been presented). Low evidence consisted of trials with zero (for third and fifth arrow) or one (for fourth arrow) prior accumulated evidence at the onset of the arrow. High evidence consisted of trials with two (for third and fifth arrow), three (for fourth arrow), or four (for fifth arrow) prior accumulated evidence at arrow onset.

**Figure 3:**
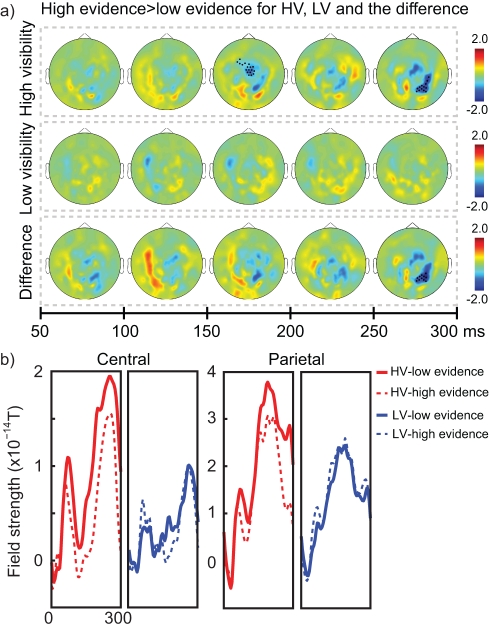
Neural markers of accumulated evidence. (A) Topographical representation of larger activity for arrows when the amount of previously accumulated evidence was low (low evidence), compared to when the amount of previously accumulated evidence was high (high evidence). Other conventions as in Figure 2. (B) Time course of central and parietal clusters. Central activity is plotted for the cluster that showed significantly reduced activity when accumulated evidence is high for HV arrows in the 150–200 ms interval. Parietal activity is plotted for the cluster that showed significantly stronger effects of accumulated evidence for HV than LV arrows in the 250–300 ms interval.

We found that when participants had high prior accumulated evidence, the newly incoming arrows evoked a smaller activity at right occipito-parietal and central sensors. Crucially, this phenomenon was significant only for HV arrows (central sensors: 150–200 ms interval, *p*_cluster_ = 0.014; occipito-parietal sensors: 250–300 ms interval, *p*_cluster_ = 0.041) (Figure 3A, top row), while there was only a non-significant trend for LV arrows in central sensors (150–200 ms interval, *p*_cluster_ = 0.077) (Figure 3A, middle row). This resulted in a significant difference between conditions over right occipito-parietal sensors (HV versus LV: 250–300 ms interval, *p*_cluster_ = 0.042) (Figure 3A, bottom row). Whereas neural responses are collapsed across arrows in this figure, Figure S2B shows that this effect was robustly observed in response to individual arrow presentations preceded by low and high evidence (a difference defined only for the third to fifth arrow, since differences in amount of accumulated evidence only arise after two arrows have been presented).

### Neural Activity Related to a Change of Evidence

**Figure 4:**
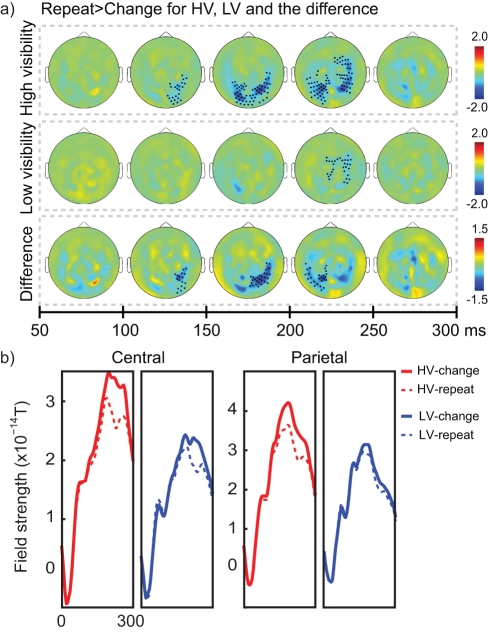
Neural markers of change in evidence. (A) Topographical representation of reduced activity for arrow stimuli that were preceded by an arrow with the same direction (“repeat”), compared to arrow stimuli which were preceded by an arrow with the opposite direction (“change”). Other conventions as in Figure 2. (B) Time course of central and parietal clusters. Central activity is plotted for the cluster that showed significant differences for both LV and HV arrows in the 200–250 ms interval. Parietal activity is plotted for the bilateral cluster that shows a significantly larger difference for HV than LV arrows in the 150–250 ms interval.

Under conditions of purely linear addition and subtraction of information, the direction of the previous arrow should not influence how the current arrow is processed. However, previous studies have described an automatic influence of repetition compared to alternation during decision-making [32], and previously we also showed a large reduction in neural activity for repeated compared to non-repeated arrows under conditions of high visibility [20]. When directly contrasting “repeat” arrows (i.e. arrows that were preceded by an arrow with the same direction) with “change” arrows (i.e. arrows that were preceded by an arrow with the opposite direction), we observed a large neural activity reduction for arrow repetitions (neural responses are collapsed across arrows). For HV arrows, this reduction was visible at occipito-parietal (100–150 ms interval, *p*_cluster_ = 0.030; 150–200 ms interval, *p*_cluster_ = 0.005; 200–250 ms interval, *p*_cluster_ = 0.022) and central (HV: 200–250 ms interval, *p*_cluster_ = 0.019) sensors (Figure 4A, top row). A similar effect was also observed for LV arrows at central sensors only (200–250 ms interval, *p*_cluster_ = 0.022) (Figure 4A, middle row), in line with earlier studies showing subliminal repetition suppression effects [9],[33]. Nevertheless, a direct comparison between both conditions shows that this suppression effect was significantly larger for HV than LV arrows (HV versus LV: 100–150 ms interval, *p*_cluster_ = 0.039; 150–200 ms interval, *p*_cluster_ = 0.015; 200–250 ms interval, *p*_cluster_ = 0.05) (Figure 4A, bottom row). Examination of the neural response to each individual “change” and “repeat” arrows (defined only for the second to fifth arrow, since the first arrow does not have a preceding arrow) shows that this effect was robustly found whenever a new arrow was presented, with no tendency to decrease with time (Figure S2C). Further, restricting the LV analysis to the poorest perceivers who scored at chance level in the six-choice discrimination task (16.7%) showed that this effect was present equally robustly for these nine “poor perceivers” (see Figure S3).

## Discussion

In a combination of behavioral and electrophysiological studies, we showed that while participants are able to accumulate evidence over time independently from the level of awareness of the evidence, there were marked differences between accumulation of low-visibility (LV) and high-visibility (HV) information, both in terms of brain activity and behavior. Although the amount of bottom-up information provided by a single HV or LV arrow was identical, as measured by priming (Figure 1K), the overall decision-making performance was much less accurate when based on LV evidence than on HV evidence (Figure 1D). More interestingly, decision-making speed was modulated by the amount of accumulated evidence, but only for HV stimuli (Figure 1E). Also, subjective confidence in decision making was markedly lower for LV than HV evidence (Figure 1H). Together, this suggests that while participants could accumulate LV evidence over time, there are qualitative differences in accumulation of evidence depending on the level of awareness of the sensory information.

We observed a strong top-down biasing effect of the amount of previously accumulated information, only for HV evidence: the impact of the last arrow stimulus on the final decision decreased linearly with the amount of previously accumulated evidence (Figure 1G). Interestingly, participants did not stop accumulating HV evidence altogether when they had accrued enough information for their decision: even when a large amount of evidence (4 units) had already been accrued for one of the two decisions, the last arrow still had an impact on the decision process, which was equally large as the impact of any of the LV stimuli. This suggests that participants did not adopt a fully “logical” or digital counting strategy (perhaps for lack of time, as arrows come in at a fast pace of one every 300 ms). Rather, on HV trials only, they attributed a weight to later arrows that was inversely related to the amount of already accumulated evidence.

These behavioral findings constrain the theoretical modeling of the task. The observed strategy is not predicted by simple linear accumulation models [34], since these would predict equal weighting of later arrows, independently of the amount of previously accumulated evidence. It is also not in line with a simple gain of accumulation from LV to HV stimuli, since this would result in overall larger weights of each arrow, but no differential modulation by time and prior accumulated evidence. Rather, the results suggest a more sophisticated mode of evidence accumulation, in which the update signal is scaled with respect to the previously accumulated evidence. This behavior arises naturally from Bayesian and sequential sampling (SPRT) models [20],[21], where evidence is only accumulated up to a bound. Beyond this bound, further evidence no longer contributes to the decision, with two consequences: (1) on average, later evidence is given a smaller weight, especially when early evidence is strong and the bound is therefore likely to be reached; (2) response time accelerates in proportion to the likelihood of reaching the bound. Both of these properties accurately characterize the participants' behavior on HV trials.

Importantly however, this modulation of evidence accumulation by prior accumulated evidence was absent for LV stimuli, where the impact of each arrow was not dependent on temporal position or prior amount of accumulated evidence. Such a purely linear accumulation of evidence is exactly what is predicted from optimal Bayesian integration, assuming that the amount of available evidence is low and therefore the accumulated amount rarely reaches threshold (see [20], Figure 2C). This hypothesis can also explain why RT remained constant on LV trials, independently of total evidence: even when five arrows point in the same direction, the total accumulation would still remain below the decision threshold on most trials, thus always requiring a forced-choice decision.

Overall, the simplest theoretical model therefore is that LV and HV trials were processed through a similar accumulation-decision pathway, yet with LV trials yielding a much lower level of evidence and therefore remaining far from decision threshold. Conversely, full awareness of the stimuli may be necessary for their accumulated evidence to reach a decision threshold which enables strategic top-down biasing of later evidence accumulation based on the past accumulation.

Magneto-encephalographic (MEG) recordings lend further support to this view. They showed that, while initial perceptual processing was identical for LV and HV evidence, there was a late divergence between LV and HV, which could be seen ∼145 ms after stimulus onset over occipital cortex (Figure 2). This late divergence between LV and HV stimuli has been observed earlier using different masking paradigms [35]–[37], and these findings are generally in good accordance with a feedback view of masking, in which initial processing in visual areas is intact but late amplification by feedback is disturbed [19],[38]–[40]. Note, however, that our data do not allow us to make firm claims about the underlying mechanisms of metacontrast masking, as different explanations have also been put forward to explain the late sensory divergence (e.g., [37]). There was also a neural difference between LV and HV stimuli over left frontal and fronto-central sensors, which became significant as early as ∼55 ms. This early frontal difference could be seen for all arrows except for the first arrow of the sequence (Figure S2A). Interestingly, there was a behavioral counterpart of this effect: whereas the first arrow had equal effects on the decision for LV and HV arrows, there were large differences in the weight of the subsequent arrows on the decision (Figure 1F). This suggests that only after the visibility of the sequence was established, on the basis of the first arrow, did participants treat the incoming information differently for LV and HV arrows. We speculate therefore that this frontal amplification may be a source of the behaviorally observed biasing effect [41].

While a change in evidence increased activity over parietal and central areas for both HV and LV evidence (albeit weaker, Figure 4), a neural influence of accumulated evidence on the processing of the current arrow was again found only for HV evidence. This MEG observation corroborates earlier behavioral and neural results [20] and suggests a neural implementation of the biasing of later information by past visible information, namely by a late (∼200–300 ms after stimulus onset) top-down modulation of sensory representations (Figure 3).

By manipulating the configuration of the mask only, we created large differences in stimulus visibility without introducing differences in stimulus strength [29], as evidenced by equal priming effects under LV and HV conditions when a single arrow was presented (Figure 1K). Given that priming was unrelated to stimulus awareness, it is quite remarkable that the accumulation of evidence was. What may underlie these differences? Direct automatic priming effects are probably mediated by fast feedforward activations, which directly influence the evolving motor decision program [10]. These feedforward activations are relatively “automatic” [35] and have been found to be unaffected by stimulus visibility [14], although they can be modulated by several top-down factors, such as attention [42],[43] and task-set [44]. In contrast, the slow accumulation of evidence over time, as probed in the present study, may require self-sustainable recurrent interactions between distant brain regions, which may only be present when participants have complete access to (i.e., full awareness of) the stimuli [19].

Previous studies have shown that subliminal information can be accumulated linearly over a few hundreds of milliseconds [13],[14],[45]–[47]. Although indirect consequences of subliminal information can be measured for several minutes [17] and up to even as long as 24 h after its presentation [18], these effects may betray a form of learning and therefore synaptic changes rather than long-lasting subliminal activation. Indeed, most priming studies reveal a fast decay of subliminal information within less than one second [48],[49]. Relative to this state of knowledge, the current study is the first to show that information from sequentially presented masked stimuli can be accumulated linearly over a long duration of more than a second. However, we also show a qualitative difference in *how* evidence is treated by the nervous system depending on the level of sensory awareness. As noted above, this qualitative difference need not imply that the processing pathway is entirely different for HV compared to LV trials. Rather, the same decision mechanism may be involved, with the main difference being that only a trickle of evidence is accumulated on LV trials, with the consequence that the decision threshold is typically not reached, therefore preventing the subsequent deployment of top-down strategies for down-weighting further incoming arrows. Indeed, our results suggest that the parietal and prefrontal regions that implement such decision making by evidence accumulation [1],[2] may integrate sensory evidence across long periods of time, whether or not the original information was above or below the threshold for conscious access, but with a much weaker signal in the latter case.

A similar conclusion was reached by Sackur and Dehaene [50] when studying sequential two-step tasks with subliminal versus visible digits. As here, a qualitative behavioral difference was seen: participants were only able to perform a chained task of addition followed by comparison when the target digits were consciously seen, although they could perform each individual computation above chance when the digits were subliminal. This difference, although qualitative, could have arisen from the fact that subliminal digits did not yield enough evidence to ever reach threshold for the first computational step of the chained task. Thus, as in the present case, the same processing chain could have been in place on both conscious and non-conscious trials, but with non-conscious stimuli providing much smaller evidence that did not allow participants to go past the first processing stage and deploy further strategies.

There has been ample speculation about the function of awareness, ranging from none whatsoever [51],[52] to enabling social communication [53]. Our results suggest a potential role of awareness in biasing information processing, namely the strategic exploitation of information in line with prior expectations and goals. This proposal fits with earlier hypotheses which link conscious access with flexible information processing, owing to the possibility of quickly circulating the conscious information to virtually all of the brain's higher level processors [54]–[57]. It also fits with a role of consciousness in enabling “meta-cognition,” the ability to introspect about self-performance, which also has been associated with high-level processing in the prefrontal cortex [58]. Here, this strategic biasing process showed clear behavioral and neural advantages: it sped up processing and reduced neural computations related to the decision process when enough evidence had already been accrued. Under conditions of severely degraded evidence (such as near-threshold or subliminal information), the most rational strategy could, however, be to give each piece of evidence equal weight [20]. Interestingly, the strategic biasing process for highly visible information may exactly be the reason why “conscious” decision-making may in some special cases actually be poorer than “unconscious” decision-making [59],[60], namely when an unbiased (equal) weighting of the evidence is required.

## Materials and Methods

### Participants

All participants in all experiments had normal or corrected-to-normal vision. The study was approved by the local institutional review board (CMO Arnhem-Nijmegen), and a written informed consent was obtained from the participants according to the Declaration of Helsinki, explicating that they agreed to participate in the MEG and behavioral experiments.

### Stimuli

The experimental stimuli in all experiments were leftward and rightward pointing arrows. Stimuli were black, presented on a grey background, and subtended visual angles of 2.0°×0.87° (see Figure S1). Stimuli were presented using a PC running Presentation software (Neurobehavioral systems, Albany, USA) and shown on a screen that was ∼75 cm away from the participant. Mask stimuli were constructed such as to either substantially reduce visibility of the stimuli (metacontrast mask), leading to low-visibility (LV) stimuli, or have only weak masking properties (pseudo mask), leading to high-visibility (HV) stimuli. Masks were identical in terms of overall luminance.

### Experiment 1: Decision-Making Task

Sixteen healthy participants (5M/11F, age range 23–35) participated in the decision-making task (640 trials) within the MEG environment. Participants were presented with sequences of five successive arrows, each of which were briefly presented (17 ms), and followed 50 ms after its onset by a mask (100 ms), and a blank (150 ms). Therefore, the stimulus onset asynchrony (SOA) between successive arrows was 300 ms. Half of the trials contained metacontrast masks (leading to low-visibility [LV] stimuli) and the other half pseudo-masks (leading to high-visibility [HV] stimuli; see Figure 1A). Each arrow sequence contained either all LV or all HV arrows. At the end of each arrow sequence, the fixation square turned green, and the participants had to decide as quickly as possible whether the predominant direction of the arrow stimuli was left or right, by pressing a button with their left or right hand. Participants had to respond within a 500 ms time window. Each trial was followed by a baseline interval, during which a red fixation square was displayed for an average duration of 2,000 ms (between 1,750 and 2,250 ms). Several days before the MEG experiment participants were invited to the lab day to practice the task (∼0.5 h). Prior to MEG data acquisition, participants engaged in an additional brief training session. During MEG data acquisition, participants engaged in 10 task blocks, each block consisting of 64 trials. Total duration of the experiment was ∼60 min. For five participants, we collected only eight task blocks, due to time constraints.

For the analysis of reaction times (RT) and responses, we discarded trials to which participants responded very early (RT<150 ms), after the reaction time cut-off (RT>500 ms) or not at all (missed trials). For the analysis of responses, we compared the proportion of left/right responses as a function of the amount of accumulated evidence for a left/right response, for HV and LV trials (Figure 1D). For reaction times, we compared the RTs as a function of accumulated evidence for HV and LV trials (Figure 1E). For the analysis of arrow impact as a function of time, we used a logistic multiple regression analysis, in order to independently estimate the effect of each arrow on the decision (Figure 1F). For the analysis of arrow influence as a function of previously accumulated evidence, we quantified the change in proportion of left/right response as a function of the direction of the last arrow, for the three levels of previously accumulated evidence (0, 2, and 4; see Figure 1B and Figure 1G). To investigate (differences in) linear trends as a function of accumulated evidence or time, we performed linear regression analysis for each participant and tested the significance of (differences in) slopes using (paired samples) *t* tests.

We recorded ongoing brain activity during Experiment 1 using a whole-head MEG with 275 axial gradiometers (VSM/CTF Systems, Port Coquitlam, British Columbia, Canada). Head localization was monitored continuously during the experiment using coils that were placed at the cardinal points of the head (nasion, left and right ear canal). The magnetic fields produced by these coils were used to measure the position of the participant's head with respect to the MEG sensor array. In addition to the MEG, the electrooculogram (EOG) was recorded from the supraorbital and infraorbital ridge of the left eye for the subsequent artifact rejection.

All data analysis was performed using the FieldTrip toolbox developed at Donders Institute for Brain, Cognition and Behaviour [61] using Matlab 7 (MathWorks, Natick, MA, USA). Data were checked for artifacts using a semiautomatic routine that helped detecting and rejecting eye blinks, muscle artifacts, and jumps in the MEG signal caused by the SQUID electronics. Subsequently, independent component analysis was used to remove any heart artifacts and eye movements not rejected by the semiautomatic routine. Finally, we low-pass filtered the data using a two-pass Butterworth filter (filter order of 6, frequency cut-off of 40 Hz). We did not apply any high-pass filter. We calculated an estimate of the planar gradient for the data analysis on the sensor level. The horizontal and vertical components of the planar gradients were calculated for each sensor using the signals from the neighboring sensors, thus approximating the signal measured by MEG systems with planar gradiometers. The planar field gradient simplifies the interpretation of the sensor-level data because the maximal signal typically is located above the source [62]. We established the significance of the differences in field strength for each experimental factor at the cluster level, using a nonparametric cluster randomization test. This test effectively controls the type I error rate in situations involving multiple comparisons (such as 275 sensors) by clustering neighboring sensor pairs that exhibit the same effect. The randomization method first identified sensors whose *t* statistics exceeded a critical value when comparing two conditions sensor by sensor (*p*<0.05, two-sided). In the second step, to correct for multiple comparisons, contiguous sensors (separated by <5 cm) that exceeded the critical value (as defined in the first step) were considered a cluster. The cluster-level test statistic was defined from the sum of the *t* values of the sensors in a given cluster. The cluster with the maximum sum was used in the test statistics. The type I error rate for the complete set of 275 sensors was controlled by evaluating the cluster-level test statistic under the randomization null distribution of the maximum cluster-level test statistic. This was obtained by randomizing the data between the two conditions across multiple participants, calculating *t* statistics for the new set of clusters. A reference distribution of cluster-level *t* statistics was created from 1,000 randomizations. The *p* value was estimated according to the proportion of the randomization null distribution exceeding the observed maximum cluster-level test statistic (the so-called Monte Carlo *p* value). MEG data analysis focused on (1) overall differences between processing of LV and HV information; (2) neural markers of accumulated evidence for LV and HV information; and (3) effects of change in evidence (i.e., repeated versus different arrow direction) for LV and HV information. In all cases, we performed statistical tests (corrected for multiple comparisons) at five intervals after the onset of the arrow stimulus (from 50–300 ms in 50 ms steps). The first 50 ms after the onset of each arrow stimulus were used as a baseline interval. This “baseline” interval was physiologically motivated, for it takes approximately 50 ms for a visual stimulus to reach the cortex [63]. The aim of this baseline procedure was to effectively remove spill-over of overall activity from the previous arrow by subtracting out the activity at the onset of the arrow stimulus. A caveat of this procedure is that the previous LV/HV arrow may lead to a late (>350 ms) difference in evoked activity, which is misinterpreted as early differential activity evoked by a later arrow. Inspection of non-baseline-corrected traces suggests that this was not the case for our data (see Figure S2A, lower panel), but this possibility can nevertheless not be conclusively ruled out. For the analysis of overall differences between LV and HV arrows, we compared activity during LV and HV arrows, averaged across all five arrow presentations. For the analysis of global effects of accumulation evidence, we compared activity for LV and HV arrows that had “low prior accumulated evidence” and “high prior accumulated evidence,” averaged across the third to fifth arrow presentation (since there is no differential amount of prior accumulated evidence until after the first two arrows are presented). Low evidence consisted of trials with zero (for third and fifth arrow) or one (for fourth arrow) prior accumulated evidence at the onset of the arrow. High evidence consisted of trials with two (for third and fifth arrow), three (for fourth arrow), or four (for fifth arrow) prior accumulated evidence at the onset of the arrow. For the analysis of the effect of change in evidence, we compared activity for LV and HV arrows that were either preceded by the same arrow (repeat) or preceded by the opposite arrow (change), averaged across the second to fifth arrow presentation (since there is no preceding arrow until after the first arrow is presented).

We also sought to establish the first time point of significant differences between LV and HV stimuli for the three clusters that showed a significant difference between stimulus types (fronto-central, parietal, and occipital clusters). We carried out *t* tests on 5 ms time intervals for the 50–300 ms interval after stimulus onset, on the difference wave between HV and LV stimuli, for each cluster. We defined the first time point of significant difference between HV and LV stimuli as the first sample in which five contiguous samples (i.e., 25 ms) showed a significant (*p*<0.05, two-tailed) difference between conditions.

### Experiment 2: Visibility Task

All participants of Experiment 1 also participated in Experiment 2, while still in the MEG environment. To test visibility of strongly and weakly masked arrows, participants engaged in a six-choice discrimination task (120 trials, 50% LV and 50% HV). Stimulus and trial timing was exactly the same as in the experimental task with the exception that after the presentation of a trial, the question “How many arrows were pointing to the left/right?” was presented. This question remained on the screen until the participant made a response, after which a new trial started. Participants had to indicate their decision by pressing one of six response buttons. Whether participants were instructed to detect right- or left-pointing arrows was counterbalanced across participants. Before administering this task, participants were told that only accuracy was important in this task, not the speed of responding. To minimize strategic guessing, participants were notified of the fact that in this task, overall, equal numbers of trials (10) of each evidence level were presented.

### Experiment 3: Confidence Task

Sixteen participants (5M/11F, age range 20–32), who did not participate in Experiments 1 and 2, took part in the confidence-rating task (512 trials). Here, we assessed the relationship between decision-making performance and subjective confidence. Stimulus parameters and timing were all identical to Experiment 1, with the exception of an additional confidence question at the end of each trial, after the participant had given his/her response. The following question was presented, 1 s after the participants' response: “How confident are you about your response?” This sentence remained on the screen until the participant made a response, after which a new trial started. Participants had to indicate the confidence in their decision by pressing one of six buttons on the keyboard (1 = “pure guess”, 6 = “100% sure”). The confidence response was unspeeded.

### Experiment 4: Masked Priming Task

All participants of Experiment 3 also participated in Experiment 4. Here we assessed the amount of priming engendered by LV and HV arrows, using a masked priming experiment (640 trials). Here, the outline of the mask also formed an arrow stimulus (see Figure S1). Participants were instructed to respond as fast as possible to the direction of the mask arrow while ignoring the preceding prime arrow. Stimulus duration was the same as in Experiment 1. Each trial was followed by a baseline interval with an average duration of 1,000 ms (between 750 and 1,250 ms).

## Supporting Information

Figure S1 Stimuli used in the experiments. Arrow stimulus. The arrow stimuli subtended a visual angle of 2.0° by 0.87° in all experiments. For Experiments 1–3, we used rectangular mask stimuli. Mask stimuli were constructed such as to either have only weak masking properties (pseudo mask), leading to high-visibility (HV) stimuli, or substantially reduce visibility of the stimuli (metacontrast mask), leading to low-visibility (LV) stimuli. Masks were identical in terms of overall luminance. For the masked priming task (Experiment 4), the outline of the mask also formed an arrow stimulus.
(EPS)

Figure S2 Time course of activity (differences) for each arrow. (A) Time course of overall differences in neural processing of low- and high-visibility arrow stimuli, for the fronto-central, parietal, and occipital cluster outlined in Figure 2. Shown are baseline-corrected traces (top panel) as well as non-baseline-corrected traces (bottom panel). A direct comparison of these shows that there is a superposition of activity for each subsequent arrow stimulus as well as an overall activity increase, which is strongest over frontal sensors, and somewhat visible over occipital and parietal sensors. By removing the baseline differences, the signal reflects the increase in activity generated by the current stimulus, over and above the activity generated by earlier arrows. (B) Time course of global effect of accumulated evidence for “low evidence” and “high evidence” arrows, for low- and high-visibility arrow stimuli, in the clusters outlined in Figure 3. (C) Time course of activity for “repeat” and “change” arrows, for low- and high-visibility arrow stimuli, in the clusters outlined in Figure 4.
(EPS)

Figure S3 Neural markers of change of evidence for nine “poor perceivers” who scored at chance level in the six-choice discrimination task. Topographical representation of larger activity (between 150 and 250 ms) for arrow stimuli that were different from their directly preceding arrows (“change”), compared to arrow stimuli which were the same direction as their directly preceding arrows (“repeat”). This activity difference was plotted for high-visibility (top row) and low-visibility (middle row) arrows, as well as the difference in activity between them (bottom row). Differences are robust and qualitatively similar to those in the whole group (see Figure 4 for whole group results).
(EPS)
