# Correction: How Awareness Changes the Relative Weights of Evidence During Human Decision-Making

**DOI:** 10.1371/annotation/10373881-f9b9-4ca9-8121-3ea8b6fc0dc5

**Published:** 2013-03-13

**Authors:** Floris P. de Lange, Simon van Gaal, Victor A. F. Lamme, Stanislas Dehaene

In the fifth sentence of the third paragraph of the "Behavioral Markers of Evidence Accumulation Over Time" section of the Results, the relevant sentence should be: We observed a monotonically increasing impact of arrows on the decision as a function of time for HV stimuli (T_15_=3.74, p=0.002), while this modulation of time was not significantly present for LV stimuli (T_15_=1.36, p=0.19), leading to a significant interaction (T_15_=2.90, p=0.011, Figure 1F).

