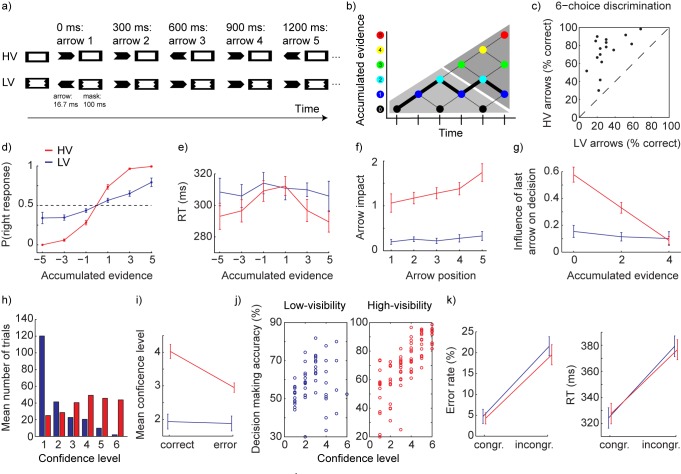# Correction: How Awareness Changes the Relative Weights of Evidence During Human Decision-Making

**DOI:** 10.1371/annotation/34c56efb-aa58-47a6-9ac0-09097219f751

**Published:** 2013-03-13

**Authors:** Floris P. de Lange, Simon van Gaal, Victor A. F. Lamme, Stanislas Dehaene

In Figure 1, Panel F showing the logistic regression analysis of arrow impact over time is incorrect due to a mistake in the analysis script. Please view the corrected Figure 1 here: 

**Figure:**